# Rapid Pharmacokinetic and Biodistribution Studies Using Cholorotoxin-Conjugated Iron Oxide Nanoparticles: A Novel Non-Radioactive Method

**DOI:** 10.1371/journal.pone.0009536

**Published:** 2010-03-04

**Authors:** Michelle Jeung-Eun Lee, Omid Veiseh, Narayan Bhattarai, Conroy Sun, Stacey J. Hansen, Sally Ditzler, Sue Knoblaugh, Donghoon Lee, Richard Ellenbogen, Miqin Zhang, James M. Olson

**Affiliations:** 1 Clinical Research Division, University of Washington, Seattle, Washington, United States of America; 2 Animal Health Shared Resources, Fred Hutchinson Cancer Research Center, University of Washington, Seattle, Washington, United States of America; 3 Neurobiology and Behavior Program, University of Washington, Seattle, Washington, United States of America; 4 Department of Material Science, University of Washington, Seattle, Washington, United States of America; 5 Department of Radiology, University of Washington, Seattle, Washington, United States of America; 6 Department of Neurosurgery, University of Washington, Seattle, Washington, United States of America; 7 Department of Pediatrics, University of Washington, Seattle, Washington, United States of America; 8 Seattle Children's Hospital, Seattle, Washington, United States of America; National Cancer Institute, United States of America

## Abstract

**Background:**

Recent advances in nanotechnology have led to the development of biocompatible nanoparticles for *in vivo* molecular imaging and targeted therapy. Many nanoparticles have undesirable tissue distribution or unacceptably low serum half-lives. Pharmacokinetic (PK) and biodistribution studies can help inform decisions determining particle size, coatings, or other features early in nanoparticle development. Unfortunately, these studies are rarely done in a timely fashion because many nanotechnology labs lack the resources and expertise to synthesize radioactive nanoparticles and evaluate them in mice.

**Methodology/Principal Findings:**

To address this problem, we developed an economical, radioactivity-free method for assessing serum half-life and tissue distribution of nanoparticles in mice. Iron oxide nanoparticles coated with chitosan and polyethylene glycol that utilize chlorotoxin as a targeting molecule have a serum half-life of 7–8 hours and the particles remain stable for extended periods of time in physiologic fluids and *in vivo*. Nanoparticles preferentially distribute to spleen and liver, presumably due to reticuloendothelial uptake. Other organs have very low levels of nanoparticles, which is ideal for imaging most cancers in the future. No acute toxicity was attributed to the nanoparticles.

**Conclusions/Significance:**

We report here a simple near-infrared fluorescence based methodology to assess PK properties of nanoparticles in order to integrate pharmacokinetic data into early nanoparticle design and synthesis. The nanoparticles tested demonstrate properties that are excellent for future clinical imaging strategies and potentially suitable for targeted therapy.

## Introduction

Nanomedicine, the application of nanotechnology in the practice of medicine, offers many advantages over conventional therapeutics. Properly developed, nanoparticles and other nano-scale therapeutics offer improved intracellular penetration, enhanced absorption into selected tissues, better pharmacokinetic (PK) properties, increased clinical efficacy and reduced toxicity [1]. Nanomedicine faces several challenges during preclinical development. Recently, the Food and Drug Administration (FDA) and the Alliance for NanoHealth (ANH) recognized seven scientific challenges in bringing nanomedicine to patients [2]. Among the seven challenges, three emphasize the importance of studying the pharmacokinetics of nanotechnology-based therapeutics. These include evaluation of nanoparticle biodistribution following systemic injection, the study of transport across biological barriers, and the development of imaging modalities for tracking the fate of nanomedicine over time.

Pharmacokinetic and biodistribution characteristics are important parameters to consider when designing and testing novel nanoparticles. In order to achieve an effective level of nanoparticles in the target tissue or tumor site, targeted nanoparticles should transition from circulating blood to the tissue of interest and bind to its molecular target as a first step in nanoparticle retention or cellular internalization. Unfortunately, many types of systemically injected nanoparticles are rapidly cleared from the blood stream by the reticuloendothelial system (RES) and the mononuclear phagocytic system (MPS) mainly through the liver, spleen, and bone marrow [1], [3] resulting in a low therapeutic index. Development of nanoparticles that avoid rapid clearance is a necessary requirement for sufficient delivery to the desired target [3], [4]. Nanoparticles with an extremely high circulation half-life should also be avoided as this may contribute to off target tissue toxicity and reduced signal-to-noise ratio due to non-specific binding.

Nanoparticle core, linker, and coating materials along with synthesis and purification techniques influence serum half-life and biodistribution [5]. Linker molecules like chitosan can sterically stabilize the corona, and prevent aggregation. Surface modification of nanoparticles with synthetic polymers like polyethylene glycol (PEG), polyvinyl alcohol (PVA), or polysaccharide can enhance solubility of hydrophobic materials, minimize non-specific binding, prolong circulation time, and enhance tumor specific targeting [6], [7]. Since chemical and physical properties of nanoparticles determine their *in vivo* fate, it is desirable to measure pharmacokinetic profiles early in the development process so that this information can be used to influence nanoparticle design and candidate prototype selection. Traditionally, pharmacokinetic studies were based on either quantification of the therapeutic agent (e.g., using HPLC and mass spectroscopy) or by radiolabeling the agent and measuring radioactivity in homogenized tissue [8], [9]. Both approaches are relatively expensive, time consuming, and outside of the expertise of many nanoparticle synthesis laboratories. Even though pharmacokinetic data is critical in making decisions regarding particle size, coating, synthesis procedures, and purification methods these studies are often conducted in the late stages of nanoparticle development due to these roadblocks. Here we describe a new, fast, and economical method for evaluating PK and biodistribution properties of nanoparticles using near infrared (NIR) based technology that allows these critical steps to be performed early in the nanoparticle development.

Some groups, including ours, incorporate near infrared fluorophore (NIRF) molecules into nanoparticle synthesis to facilitate intra-operative visualization of targeted cells or tissues [10], [11]. We capitalized on this reporter molecule to assess serum half-life and biodistribution of a chitosan-conjugated iron oxide core nanoparticle. This nanoparticle serves as a platform to deliver targeted anticancer therapeutics and as an imaging agent for visualizing tumors in mice [12]. The results suggest potential utility of these nanoparticles for future medical applications.

This paper describes the development of a new non-radioactive method for assessing serum half-life, biodistribution and *in vivo* stability using the NIRF, Cy5.5. This novel method provides 21-micron histological resolution for identifying populations of cells that bind to the nanoparticles. We demonstrate this new technique by analyzing the tissue distribution and serum half-life of a new chitosan-conjugated nanoparticle.

## Results

### Serum Half-Life of Nanoparticles

**Figure 1:**
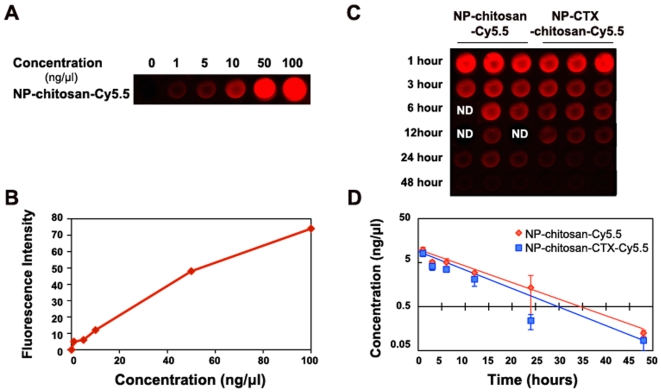
Serum half life of nanoparticles. (A and B) Standard curve generated by quantifying intensity of known concentrations of NP-chitosan-Cy5.5 or NP-CTX-chitosan-Cy5.5. (C and D) Measured fluorescence intensity of nanoparticles in serum. Each data point represents the mean fluorescence intensity integrated above the baseline. This baseline subtraction avoids systematic errors from underlying autofluorescence. Error bars represent standard errors of the mean. The curve indicates an exponential decay curve fit to the data (n = 3 mice per time point).

For assessment of serum half-life, we focused on a reproducible, quantitative assay that utilized the NIRF dye, Cy5.5, which was incorporated into the nanoparticle. Blood was collected from mice at 1, 3, 6, 10, 24, and 48 hours after injection of 200 µl of 1 mg/ml of NP-chitosan-CTX-Cy5.5 or the non-targeted control, NP-chitosan-Cy5.5. Blood was centrifuged and the plasma was collected for analysis. The blood plasma was added to a 96 well clear bottom plate and scanned using the Odyssey scanner. The Cy5.5 signal could be readily detected and quantified in small volume blood samples using a 96 well format NIR fluorescence scanner as described in the Methods section. NP-chitosan-CTX-Cy5.5 exhibited a longer circulation time than NP-chitosan-Cy5.5 (Figure 1). Exponential decay analysis of NP-chitosan-CTX-Cy5.5 revealed an elimination half-life of 8 hours compared to 7 hours for NP-chitosan-Cy5.5.

### Biodistribution

Radioactivity-based pre-clinical biodistribution studies typically fail to show anatomic resolution that would distinguish signal differences among subpopulations of cells within a tissue. Likewise, whole organ biophotonic imaging for fluorescence-labeled therapeutics also lacks microscopic resolution and further suffers from the fact that the quantitative data from whole organs is dramatically affected by size and shape of the organ. After demonstrating that the Cy5.5 fluorescent signal is retained after freezing (Figure S1), we developed an assay in which a NIR fluorescence scanner was used to quantitatively assess Cy5.5 signal at 21 micrometer resolution.

**Figure 2:**
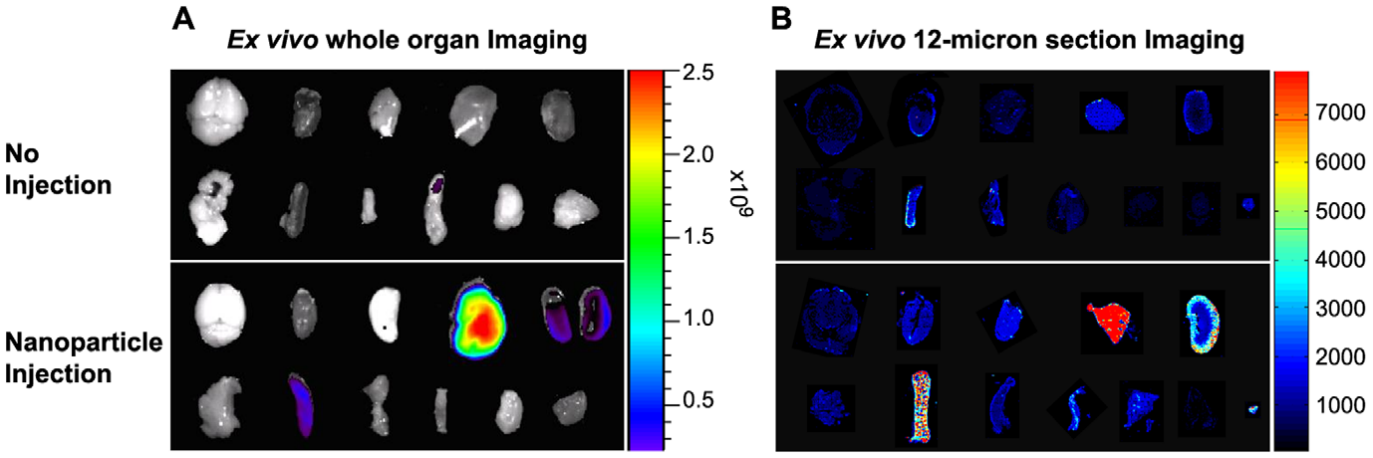
Ex vivo NIRF imaging of each organ. Fluorescence image of whole organs in non-injected (top) or NP-CTX-Chitosan-Cy5.5 injected (bottom) animals. Images were acquired six hours post injection. (A) Mice were injected 200 µl of 1 mg/ml. Six hours after injection, the animals were euthanized and the organs were collected. *Ex vivo* fluorescence images of whole organ was obtained using the Xenogen imaging system. The spectrum gradient bar corresponds to the fluorescence intensity unit p/sec/cm^2^/sr. (B) Fluorescence image of 12-micron sections obtained using the Odyssey imaging system. The spectrum gradient bar corresponds to relative fluorescent level. (Top row: brain, heart, lung, liver, and kidney. Bottom row: pancreas, spleen, small intestine, colon, gonad, muscle, and bone marrow) Bone marrow is only shown with the Odyssey scanner.

Mice were injected through the tail vein with 200 µl of 1 mg/ml NP-chitosan-Cy5.5 or NP-chitosan-CTX-Cy5.5. Whole organs were removed 6, 24, or 48 hours after injection and scanned using the IVIS-100 imaging system (Figure 2A). All tissue was frozen in OCT then sliced in 12-micron sections and mounted on glass slides. The slides were scanned on the Odyssey NIR scanner and images were obtained using the 700 nm channel (Figure 2B).

**Figure 3:**
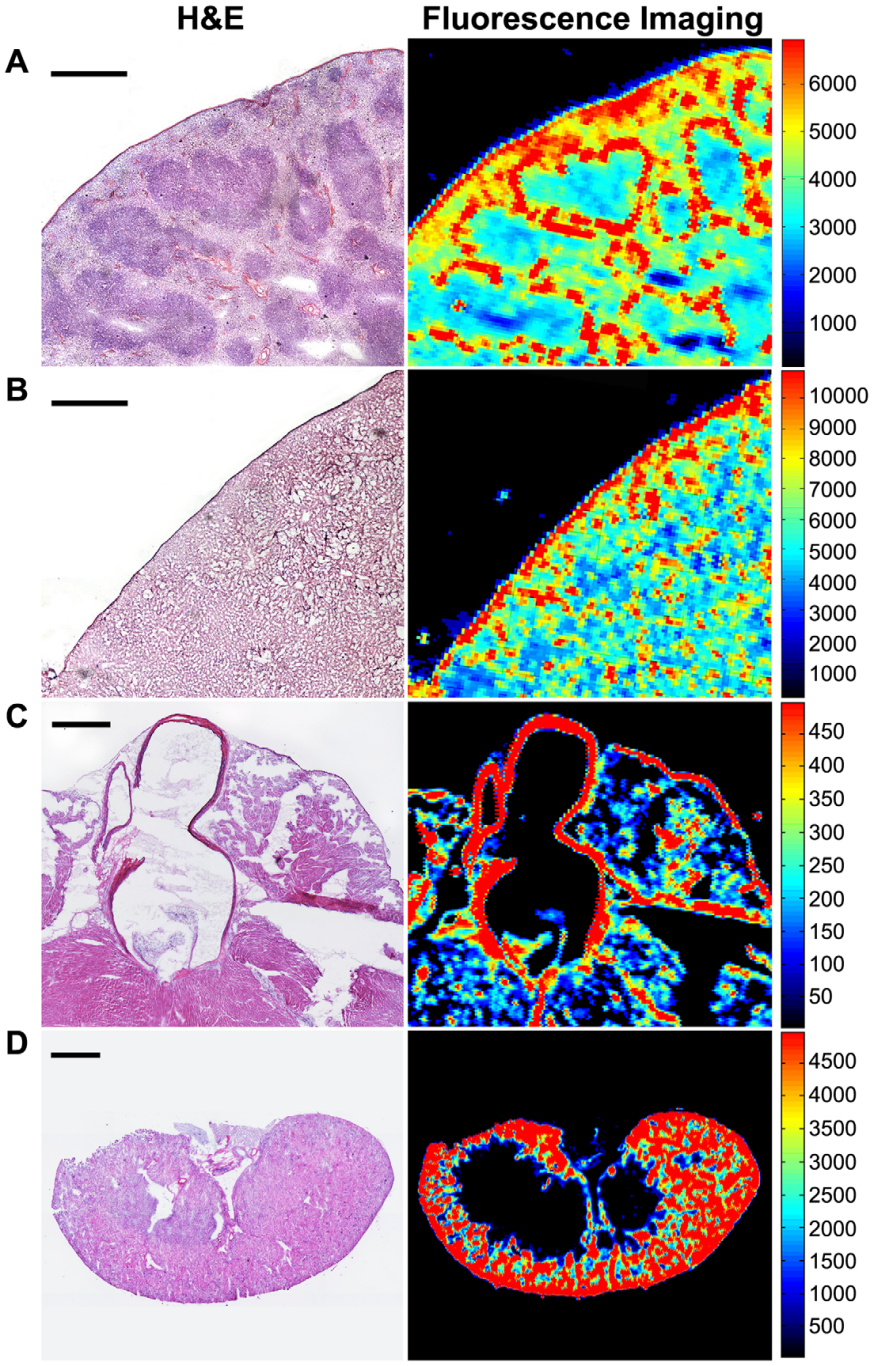
Comparison of H&E staining and high-resolution NIRF fluorescence imaging. (A) In spleen, NIRF signal was observed in large cells within the white pulp (bar, 500 µm). (B) In liver, the pattern was less obvious, but clearly heterogeneous (bar, 500 µm). (C) In heart, the muscle walls of the atria and ventricle showed no signal above background but the walls of the aorta showed significant signal (bar, 50 µm). (D) In kidney, high NIRF signal was found in the renal cortex (bar, 1 mm). All images were taken of tissues that were harvested 24 hours post nanoparticle injection.

The slice assay revealed, with anatomic resolution, small areas within certain organs that bind the conjugate that would be missed by methods that involved homogenization or whole organ imaging. For example, signal was present in the wall of the aorta in heart slices (Figure 3C), but not evident on whole organ imaging (Figure 2). While the amount of signal is small, the data show the capability of the method to detect small foci of NIRF-positive cells within an organ that otherwise appears to have minimal signal.

Consistent with whole-organ biophotonic images, significant localization was seen in slices from the tissues of the RES (liver, spleen, and bone marrow) as well as the kidneys (Figure 2 and 3). Histological analysis revealed that binding was not uniform in these tissues. The binding patterns in liver and spleen suggested macrophage uptake (Figure 3). High concentration of nanoparticles was revealed in the spleen white pulp. In kidney, the signal localized to the renal cortex.

**Figure 4:**
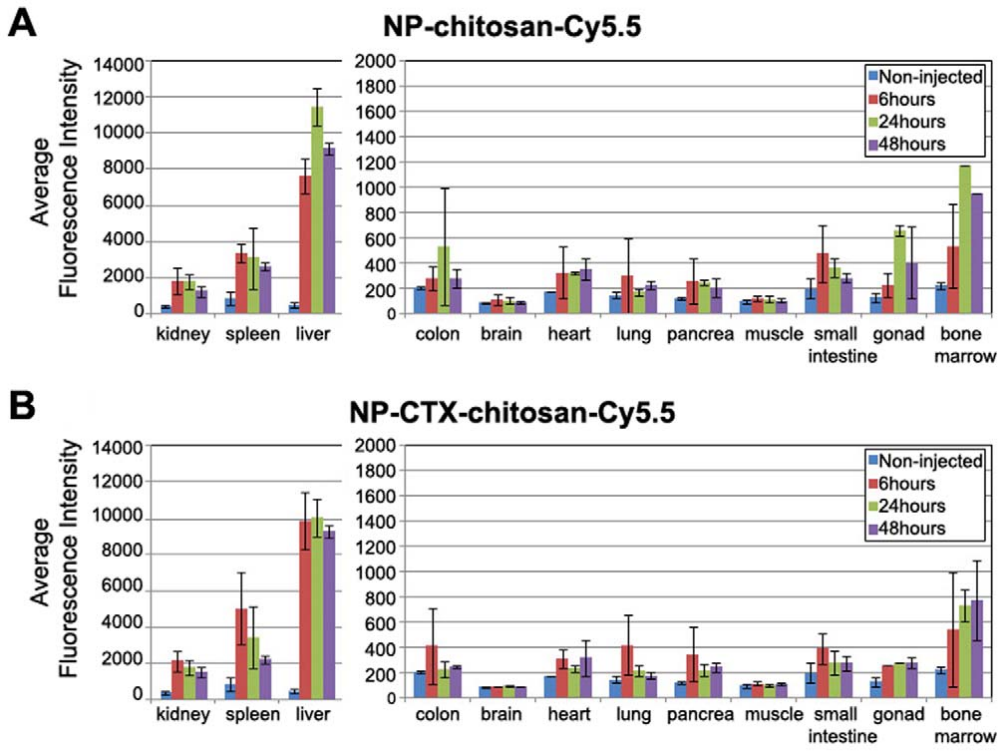
Biodistribution of NP-chitosan-Cy5.5 and NP-CTX-chitosan-Cy5.5 nanoparticles. Relative fluorescence intensity was determined using the Odyssey scanner. Bars represent tissue from animals that were not injected with nanoparticles (blue), or injected with nanoparticles and harvested 6 hours (red), 24 hours (green), or 48 hours (purple) after injection. Bars represent the average of 3 animals for each nanoparticle at each time point. The error bars are standard deviation from the mean. (A) *Ex vivo* biodistribution of NP-chitosan-Cy5.5 non-targeted nanoparticle. (B) *Ex vivo* biodistribution of NP-CTX-chitosan-Cy5.5 targeted nanoparticle.

Similar to most nanoparticles, tissue binding was higher in spleen and liver than other tissues. In spleen and liver, NIRF signal decreased slightly between hour 6 and hour 48 (Figure 4). With the exception of bone marrow, in which 4–5 fold increase in signal over background was observed, the signal in all other whole organs was statistically indistinguishable from background. There was no significant difference in normal tissue biodistribution between untargeted and chlorotoxin-targeted nanoparticles indicating that the targeting peptide influences specific binding to tumors [12], [13], but does not affect non-specific binding to normal tissue (Figure 4).

### Nanoparticle Stability

**Figure 5:**
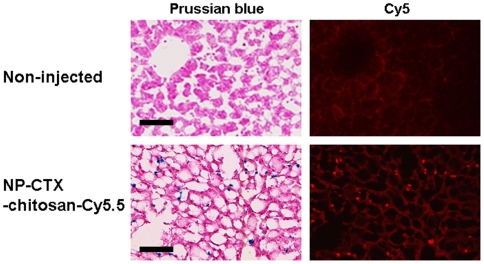
Co-localization of iron oxide nanoparticle core and Cy5 signal in liver. No iron uptake or Cy5 fluorescent signal was detected in non-injected control tissue (top row). Co-localization of Prussian blue/hemotoxylin staining and Cy5 signal from the same tissue section suggest that the nanoparticles remain stable *in vivo* (bottom row) (bar, 50 µm).

To analyze the stability of nanoparticle suspensions against agglomeration within the biological system, nanoparticles were kept in phosphate buffered saline (PBS) of physiologic pH. No apparent size change was observed after incubation of nanoparticles in media for several months. Nanoparticles were found to be stable with no sign of aggregation or loss of functionality [12]. The stability of the Cy5.5-nanoparticle-nanoparticle bond upon storage *in vivo* was confirmed by Prussian-blue staining for iron oxide nanoparticle core and Cy5 fluorescent signal in the liver. Co-localization of Prussian blue staining and Cy5 fluorescent indicate that Cy5.5 fluorophores remain attached to iron oxide nanoparticle, confirming the stability of nanoparticles *in vivo* (Figure 5).

### Toxicity Studies

Guided by the biodistribution data, tissues were carefully evaluated for evidence of nanoparticle toxicity. Mice received a single intravenous injection of 200 µl of 1 mg/ml of NP-chitosan-Cy5.5 or NP-chitosan-CTX-Cy5.5. All mice were evaluated twice weekly for the duration of the studies for clinical symptoms of toxicity. None were observed. Seven days after the injection, tissue was removed and fixed in 10% formalin. A veterinary pathologist reviewed Hematoxylin and Eosin (H&E)-stained tissue sections from liver, spleen, kidney, lung, heart, colon, skeletal muscle, ovary/testes, small intestine, and brain of mice injected with control or CTX-targeted chitosan-conjugated nanoparticles (data not shown). Despite relatively high levels of nanoparticles in liver, spleen, and bone marrow, no significant lesions were observed. In kidneys, mild multifocal tubular abnormalities were observed in subsets of both nanoparticle injected and non-injected groups, leading to the conclusion that these mild differences in histological appearance were not related to nanoparticle injection. None of the other tissues had abnormal findings. Overall, the toxicity studies indicate that the nanoparticles were well tolerated and that renal function and renal pathology should be closely monitored if the non-targeted nanoparticles are advanced toward clinical use.

## Discussion

**Figure 6:**
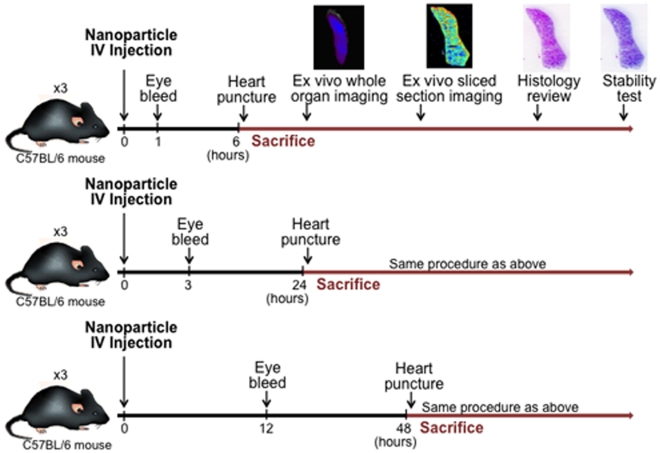
Rapid and easy method to generate key pharmacokinetic data in the early stages of nanoparticle development. Our approach minimizes the number of mice required to evaluate nanoparticle pharmacokinetic properties. Serum half-life, whole organ biodistribution, biodistribution at cellular level up to 12 µm resolution, histological and *in vivo* stability analysis can be performed from the same set of mice that are used to assess nanoparticle targeting to cancer cells or other targets.

The goal of our approach is to provide rapid and easy monitoring of nanoparticle distribution. In the early stages of nanoparticle development, when decisions are being made about materials, coatings, synthetic processes and purification, key PK data can be generated in the same mice that are utilized to assess nanoparticle targeting to cancer cells or other targets. The stability of nanoparticles is often studied in biological media such as PBS and Dulbecco's modified Eagle's medium (DMEM) with 10% of fetal bovine serum (FBS) [14]. However, nanoparticles in solutions with physiological salt concentration, pH and temperature cannot fully recapitulate different types of cells or immune system in biological system. Therefore, we focused on evaluating nanoparticles pharmacokinetic *in vivo*. In this setting, we found that Cy5.5 signal perfectly matched Prussian Blue staining for the iron oxide nanoparticles, indicating exceptional *in vivo* stability. The PK data can be utilized to inform development and make “go, no-go” decisions. Importantly, serum half-life, biodistribution, and stability are all evaluated in the same mice, which reduces the number of research animals utilized (Figure 6) and all data is generated on a single piece of equipment, which reduces capital equipment costs.

The serum half-life of NP-chitosan-CTX-Cy5.5 and NP-chitosan-Cy5.5 is 7–8 hour, which is within the ideal therapeutic range. We attribute the prolonged serum half-life to the PEG surface coating with chitosan linker, which was designed to reduce nanoparticle agglomeration and the rate of nonspecific phagocytosis of the nanoparticles by reticuloendothelial cells. Nanoparticle serum levels in the first 24 hours, coupled with the biodistribution data, indicate that both liver and kidney are involved in nanoparticle elimination. The serum half-life of the tested nanoparticles was in the range that is generally considered optimal.

Biodistribution studies revealed expected localization in kidney, spleen, and liver for both targeted and non-targeted nanoparticles. High uptake by mononuclear phagocytic system in the liver and spleen are one of the greatest challenges of using nanoparticles for tumor targeting. Our new approach allows quantitative measure of nanoparticles distributed in deep tissues of MPS-rich organs with anatomical resolution.

The biodistribution findings have different implications for therapeutics versus imaging. For imaging tumor foci, the primary concern is the signal in tumor compared to adjacent normal tissue and it does not matter whether other organs have higher signal than the tumor unless they are potential sites of metastases or might obscure visualization of the tumor area by emitting bright signal. In contrast, targeted therapeutics depends on either having higher targeting to tumor tissue than all other organs that might be adversely affected by the therapeutic payload or choosing to deliver a therapy that does not adversely affect organs that sequester or excrete nanoparticles nonspecifically.

NIR imaging enables relatively quick and simple measurements of serum half-life, biodistribution and stability. These data can be incorporated into early nanoparticle synthesis decisions as well as more detailed documentation required for nanoparticles that are submitted to the FDA for human clinical trials. The data obtained from NIR fluorescence imaging provides sensitive, specific, and real-time pre-clinical information that complements efficacy studies focused on tumor visualization.

## Materials and Methods

### Nanoparticle Synthesis and Characterization

Nanoparticles were synthesized as described previously [12]. Transmission electron microscopy (TEM) has shown the iron oxide cores with a mean diameter of 7 nm. BCA protein quantification and fluorescence quantification determined a yield of 16.2 CTX peptides and 1.5 fluorophores per nanoparticles on average.

### Serum Half-Life

All animal studies were conducted in accordance with Fred Hutchinson Cancer Research Center's Institute of Animal Care and Use Committee (IACUC) approved protocols as well as with federal guidelines. 3–5 month old mice on a C57BL6 background (Charles River Laboratories, Inc.) were injected through the tail vein with 200 µl of 1 mg/ml targeted NP-CTX-chitosan-Cy5.5 nanoparticle (n = 3) or non-targeted NP-chitosan-Cy5.5 control nanoparticles (n = 3). At six different time points after injection, 1, 3, 6, 12, 24, and 48 hours, blood was collected by retro-orbital eye bleed or terminal heart puncture. Because of limitations on the amount of blood that can be drawn from each animal, no animal was used for more than two time points. Blood samples were drawn from three independent mice for each time point and frozen at −80°C until analysis. Samples were thawed at room temperature for 30 minutes prior to analysis. Whole blood was spun using a benchtop centrifuge for 5 minutes at 10,000 rpm to separate the plasma. 50 µl of plasma was then added to a 96 well clear bottom plate. The plate was scanned on the Odyssey NIR fluorescence imaging instrument (LI-COR, Lincoln, NE) using the 700 nm-channel (λ_exc_ = 685 nm with λ_em_ = 705 nm) to measure Cy5.5 fluorescence signal. Concentration of nanoparticles was interpolated from the NP-chitosan-Cy5.5 fluorescence standard curve. A separate study was done in advance to ensure that fluorescent signal was not modified by freezing (Figure S1).

### Biodistribution of Nanoparticle

Animals were injected via tail vein with 200 µl of 1 mg/ml NP-CTX-chitosan-Cy5.5 targeted nanoparticle, or NP-chitosan-Cy5.5 non-targeted control nanoparticles. Three additional non-injected animals were included as controls. 6, 24, or 48 hours after injection (n = 3) the animals were euthanized and tissues were dissected from twelve different organs: kidney, spleen, liver, colon, brain, heart, lung, pancreas, muscle, small intestine, gonads, and bone marrow. Bone marrow was extracted by flushing the bone marrow cavity with phosphate-buffered saline (PBS) then centrifuged to pellet the marrow. Each tissue was imaged using the IVIS-100 (Xenogen Co., Alameda, CA). Tissues were then embedded in OCT and kept frozen at -80°C until needed. The frozen tissues were sliced in 12 µm thick sections and mounted onto glass slides. The tissue sections were thawed at room temperature for 30 minutes and the fluorescence intensity was measured using the Odyssey fluorescence scanner at a resolution of 21 µm. The images were analyzed with the public-domain ImageJ software (US National Institutes of Health, Bethesda, MD). The average fluorescence intensity was determined for each tissue type using the same threshold settings (low threshold: 400, high threshold: 20,000). Data are reported as the average channel fluorescence of the tissue, given as relative units after background subtraction. For visual illustrations of fluorescence signals, color maps are generated using Matlab (Mathworks, Natick, MA).

### Nanoparticle Stability

Colloidal stability of nanoparticles was evaluated by suspending the nanoparticles in PBS media of physiologic condition (pH 7.4, 37°C) for several months. For histology and fluorescence imaging analysis, 12-µm-thick sections were prepared from the same OCT embedded frozen sample as above. Fluorescence imaging of side-by-side non-injected controls and injected liver was performed in the Cy5 channel (λ_exc_ = 620–650 nm with λ_em_ = 680–710 nm) using a TissueFax-System (TissueGnostics, Vienna, Austria). The same tissue sections were stained with Prussian blue and nuclear fast red counterstain for visualizing iron nanoparticles. Sections were then imaged with TissueFax.

### Toxicity Studies

Additional Mice were injected with 200 µl of 1 mg/ml NP-CTX-chitosan-Cy5.5 (n = 3) or NP-chitosan-Cy5.5 (n = 3) via the tail vein. Seven days after the injection the following organs were harvested and fixed in 10% formalin: brain, heart, lung, liver, spleen, kidney, pancreas, gonad, small intestine, colon, and muscle. The tissues were paraffin embedded and Hemotoxylin and Eosin stained according to standard protocols. Each tissue was analyzed by a veterinary pathologist.

## Supporting Information

Figure S1 Freezing effects on NIRF signal. The stability of infrared signal during the process of freezing was addressed in a group of mice receiving free Cy5.5 dye and CTX-Cy5.5. Mice were injected via lateral tail vein with 100 µl of either Cy5.5 or CTX-Cy5.5. 24 hours post-injection the animals were euthanized and the kidneys removed. One kidney was homogenized in 1 ml of PBS, 30 µl was added to a 96 well plate and the NIRF signal was analyzed using the Odyssey imaging system before and after the freezing process.
(0.06 MB TIF)
