# Correction: Rapid Pharmacokinetic and Biodistribution Studies Using Chlorotoxin-Conjugated Iron Oxide Nanoparticles: A Novel Non-Radioactive Method

**DOI:** 10.1371/annotation/fe02e23b-5168-4586-ba49-d0e0286f4ad3

**Published:** 2010-03-09

**Authors:** Michelle Jeung-Eun Lee, Omid Veiseh, Narayan Bhattarai, Conroy Sun, Stacey J. Hansen, Sally Ditzler, Sue Knoblaugh, Donghoon Lee, Richard Ellenbogen, Miqin Zhang, James M. Olson

There was an error in the title of this article. The correct title is: Rapid Pharmacokinetic and Biodistribution Studies Using Chlorotoxin-Conjugated Iron Oxide Nanoparticles: A Novel Non-Radioactive Method. The correct citation is: Lee MJ-E, Veiseh O, Bhattarai N, Sun C, Hansen SJ, et al. (2010) Rapid Pharmacokinetic and Biodistribution Studies Using Chlorotoxin-Conjugated Iron Oxide Nanoparticles: A Novel Non-Radioactive Method. PLoS ONE 5(3): e9536. doi:10.1371/journal.pone.0009536

