# Correction: Rapid Pharmacokinetic and Biodistribution Studies Using Cholorotoxin-Conjugated Iron Oxide Nanoparticles: A Novel Non-Radioactive Method

**DOI:** 10.1371/annotation/aab4514c-8393-4f1c-a482-fd2008da36ac

**Published:** 2010-04-30

**Authors:** Michelle Jeung-Eun Lee, Omid Veiseh, Narayan Bhattarai, Conroy Sun, Stacey J. Hansen, Sally Ditzler, Sue Knoblaugh, Donghoon Lee, Richard Ellenbogen, Miqin Zhang, James M. Olson

The following statement was missing from the Funding section. "This study was supported by grant R01CA135491, 'Chlorotoxin as a Targeting Agent for Cancer Therapies.'"

