# Supplementary figures and images for: Rapid Pharmacokinetic and Biodistribution Studies Using Cholorotoxin-Conjugated Iron Oxide Nanoparticles: A Novel Non-Radioactive Method
Source: PLoS One. 2010 Mar 4;5(3):e9536. doi: 10.1371/journal.pone.0009536 (PMC2832013; doi:10.1371/journal.pone.0009536)

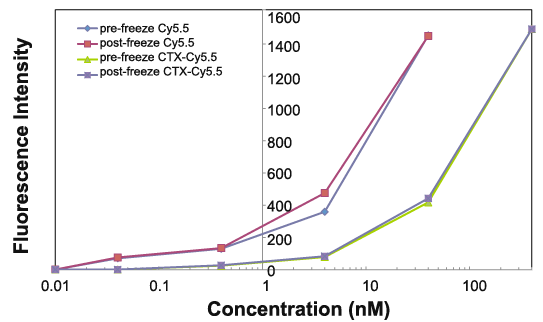

Supplement: Figure S1 — Freezing effects on NIRF signal. The stability of infrared signal during the process of freezing was addressed in a group of mice receiving free Cy5.5 dye and CTX-Cy5.5. Mice were injected via lateral tail vein with 100 µl of either Cy5.5 or CTX-Cy5.5. 24 hours post-injection the animals were euthanized and the kidneys removed. One kidney was homogenized in 1 ml of PBS, 30 µl was added to a 96 well plate and the NIRF signal was analyzed using the Odyssey imaging system before and after the freezing process. (0.06 MB TIF) [file pone.0009536.s001.tif]
